# Single-Stage Combined Embolization and Structural Allograft Reconstruction for Proximal Humerus Aneurysmal Bone Cysts in Children

**DOI:** 10.3390/children13050591

**Published:** 2026-04-24

**Authors:** Maximilian Leiblein, Johannes Frank, Ingo Marzi, Katharina Sommer, Katrin Eichler, Thomas Vogl, Nils Wagner

**Affiliations:** 1 Department of Trauma Surgery and Orthopedics, University Hospital, Goethe University Frankfurt, 60590 Frankfurt am Main, Germany; 2 Institute for Diagnostic and Interventional Radiology, University Hospital, Goethe University Frankfurt, 60590 Frankfurt am Main, Germany

**Keywords:** aneurysmal bone cyst, proximal humerus, pediatric, selective arterial embolization, allograft reconstruction, fibular graft, bone tumor

## Abstract

Background: Aneurysmal bone cysts (ABCs) of the proximal humerus in children are rare, locally aggressive lesions associated with substantial recurrence rates and risk of structural instability. Conventional treatment by curettage and bone grafting is often limited by recurrence, while selective arterial embolization (SAE) alone may not provide sufficient structural support. This study evaluates a single-stage treatment strategy combining embolization and structural reconstruction to address both the vascular and mechanical components of the disease. Methods: A retrospective analysis was performed on 12 pediatric patients (median age 9 years) with proximal humerus ABCs treated between 2020 and 2024. All patients underwent a standardized single-stage protocol consisting of preoperative SAE, intralesional resection with high-speed burr, and reconstruction using an allogeneic fibula graft combined with cancellous bone augmentation. Radiological consolidation, recurrence, and functional outcomes were assessed. Associations between prior surgery, cyst size, and recurrence were analyzed. Results: Primary consolidation was achieved in 75% of patients, with an overall healing rate of 91.7% after secondary interventions. Recurrence occurred in 16.7% of cases and was significantly associated with prior surgical treatment (*p* = 0.045). No significant correlation was found between cyst size and recurrence (*p* = 0.151). At final follow-up (median 8.5 months), all patients demonstrated complete healing according to the modified Neer classification following completion of treatment. Functional outcomes were favorable, with 91.7% of patients regaining full range of motion and no neurovascular complications observed. Conclusions: The presented single-stage approach combining SAE, intralesional resection, and structural allograft reconstruction addresses both the vascular supply and mechanical instability of proximal humerus ABCs. This strategy demonstrated high healing rates and favorable functional outcomes, with acceptable recurrence rates in this cohort while avoiding donor site morbidity. It represents a practical and effective treatment concept for this rare pediatric condition.

## 1. Introduction

Aneurysmal bone cysts (ABCs) are benign but locally aggressive lesions characterized by blood-filled septate cavities. Due to their destructive nature, they frequently lead to pathological fractures of the affected bone.

ABCs account for about 1% of all bone tumors and predominantly occur in children and adolescents, with 70% of affected patients being between 5 and 20 years [1,2]. The most commonly affected sites include the metaphysis of long bones, as well as the spine and pelvis [3].

The incidence of ABCs is estimated at 0.14 per 100,000 per year. However, data regarding gender distribution remain inconclusive [4].

The exact etiology of ABCs is not fully understood. The current literature suggests a multifactorial origin, including primary neoplastic conditions, secondary responses such as arteriovenous malformations, and genetic predisposition [1]. ABCs may occur either de novo (primary ABCs) or secondary to other bone conditions [5]. The underlying pathophysiology remains a topic of discussion. While earlier theories suggested that ABCs result from a reactive process due to increased venous pressure or arteriovenous shunting [6,7], more recent studies indicate a genetic component. Specifically, primary ABCs have been associated with a USP6 (ubiquitin-specific protease 6) gene fusion, leading to increased USP6 expression and activation of matrix metalloproteinases (MMPs) via NFkB [4,8,9]. In contrast, secondary ABCs do not exhibit USP6 rearrangements [9]. Despite these insights, no targeted therapy addressing USP6 overexpression has been reported.

Clinically, patients might present with localized symptoms such as pain, tenderness, and swelling, particularly when an extremity is involved [1]. As the disease progresses, pathological fracture may occur.

Various treatment strategies have been proposed, including curettage, radiotherapy, phenol injections, and others. In cases requiring radical treatment, en-bloc resection followed by reconstruction with vascularized or non-vascularized fibular graft reconstruction is described [10]. However, lesions at the proximal humerus pose a particular challenge, as they may compromise the growth plate and increase the risk of growth disturbances, potentially leading to limb length discrepancy.

Local recurrence rates of ABCs remain high, reported in up to 50% of patients [11,12]. While radical resection reduces recurrence, it also carries the risk of significant functional impairment and life-long problems. As an alternative, selective arterial embolization (SAE) of the feeding vessel—either as a standalone treatment or in combination with surgery—has been discussed [13].

The aim of this study is to evaluate a single-stage treatment strategy that combines embolization and structural reconstruction to address both the vascular supply and mechanical instability of proximal humerus ABCs. To our knowledge, this is one of the first studies to combine preoperative embolization with structural allograft reconstruction in a standardized single-stage protocol for proximal humerus ABCs.

## 2. Materials and Methods

### 2.1. Treatment Concept

#### 2.1.1. Diagnostics

**Figure 1:**
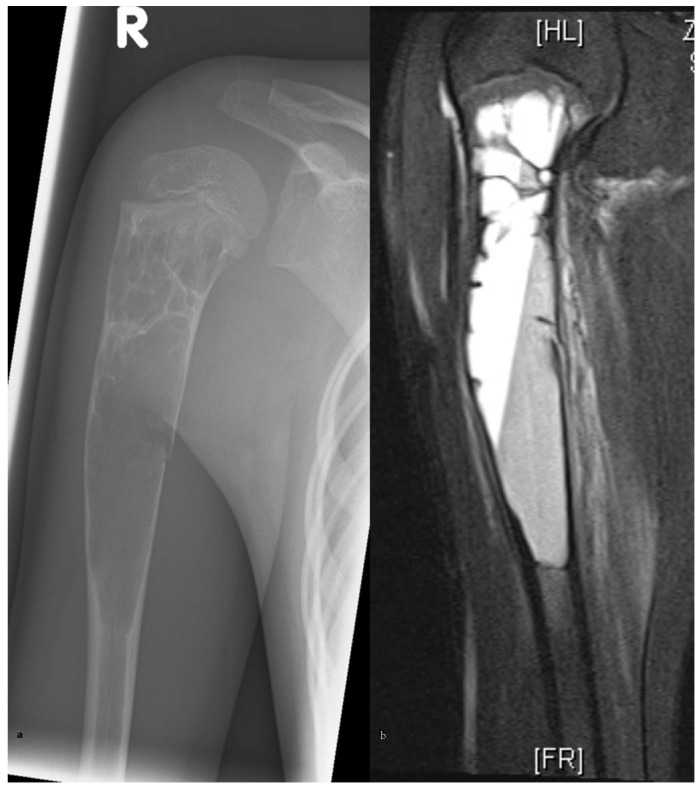
X-ray of the proximal humerus showing a bone cyst with a pathological fracture (**a**). Contrast-enhanced MRI displaying the characteristic fluid–fluid level (**b**).

A thorough clinical examination, including neurological assessment, is mandatory. Standard radiographic imaging with conventional X-rays in two planes is recommended as the initial diagnostic tool (Figure 1a).

Since ABCs are typically supplied by one or more arterial feeders, we recommend contrast-enhanced MRI angiography to confirm diagnosis (Figure 1b), differentiate between juvenile and aneurysmal bone cysts, and identify the feeding vessel. In most cases, the feeder artery arises from the circumflex humeral artery.

#### 2.1.2. Interventional Angiography

**Figure 2:**
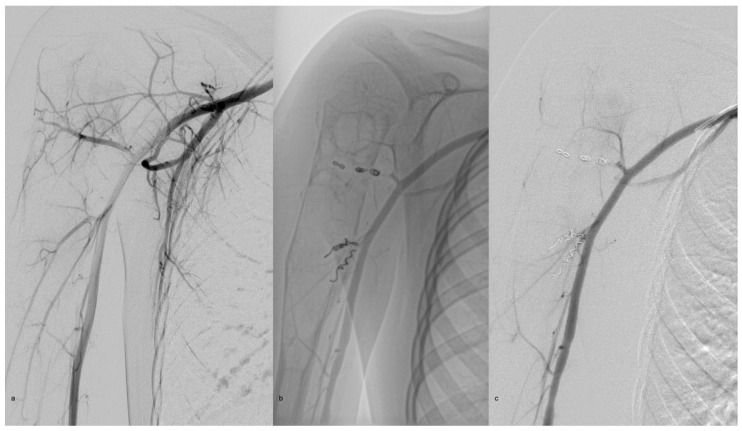
Angiographic identification of the feeding arteries (**a**) and embolization of the feeding vessels using coils (**b**,**c**).

In cases with identified feeder, preoperative interventional angiography is performed immediately before surgery, with the patient already under general anesthesia. The identified feeding artery is embolized using coils to reduce intraoperative blood loss and recurrence rate (Figure 2).

#### 2.1.3. Surgical Protocol

**Figure 3:**
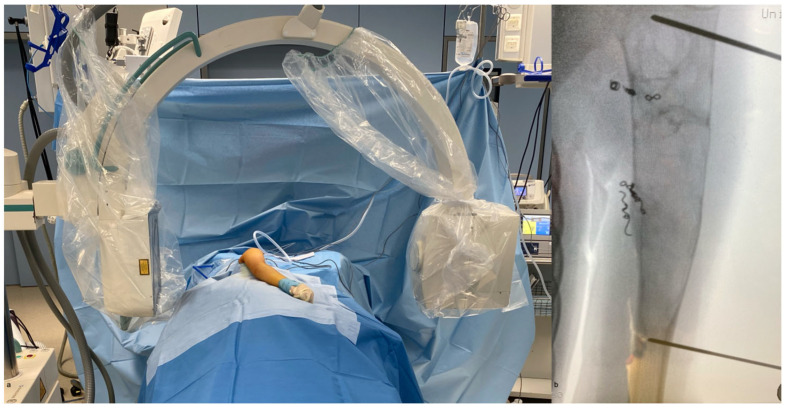
Operative setup with the patient in lateral decubitus position and the C-arm positioned for intraoperative imaging (**a**). Marking of the lesion’s extension using K-wires (**b**).

The patient is placed in a lateral decubitus position on the contralateral side, with the affected arm left free for manipulation within the sterile field. The C-arm is positioned behind the patient for intraoperative imaging (Figure 3a).

**Figure 4:**
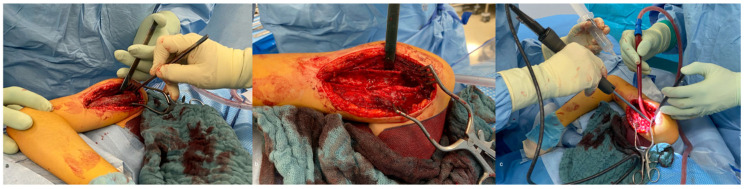
Intraoperative view on the cyst before osteotomy (**a**). Surgical site after excision of the tumorous tissue (**b**). Debridement of the lesion using a high-speed burr (**c**).

First, the extension of the lesion is marked under fluoroscopic guidance, and 1.6 mm K-wires are inserted at the proximal and distal margins of the cyst (Figure 3b). Following, a standard deltopectoral approach to the proximal humerus is performed. After exposing the humerus, the periosteum is incised and preserved to serve as a covering layer at the end of the procedure. A cortical window is created by first drilling multiple perforations along the planned osteotomy line, and the osteotomy is then completed with a chisel (Figure 4a). The tumorous tissue is completely excised using a high-speed burr (e.g., Acromionizer burr, Stryker) (Figure 4b,c). The resected tissue is collected for histopathological examination. Subsequently, the lesion is thoroughly irrigated to remove residual debris.

**Figure 5:**
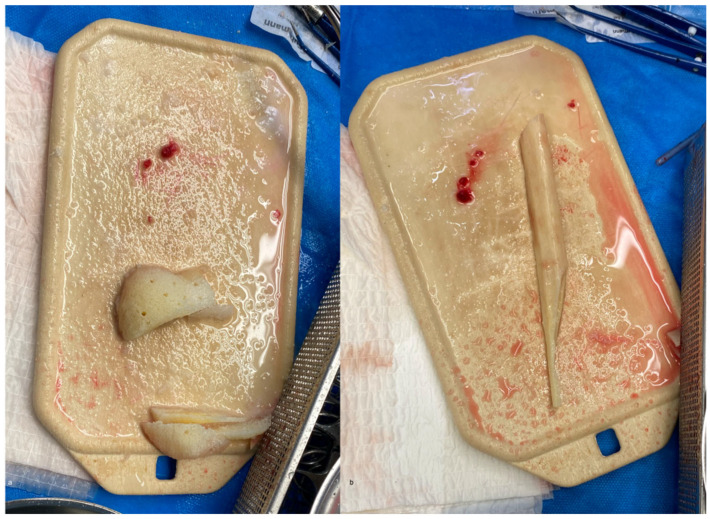
Preparation of the allogeneic femoral head (**a**) and fibula (**b**). Note the wedge-shaped distal end for optimal fit.

**Figure 6:**
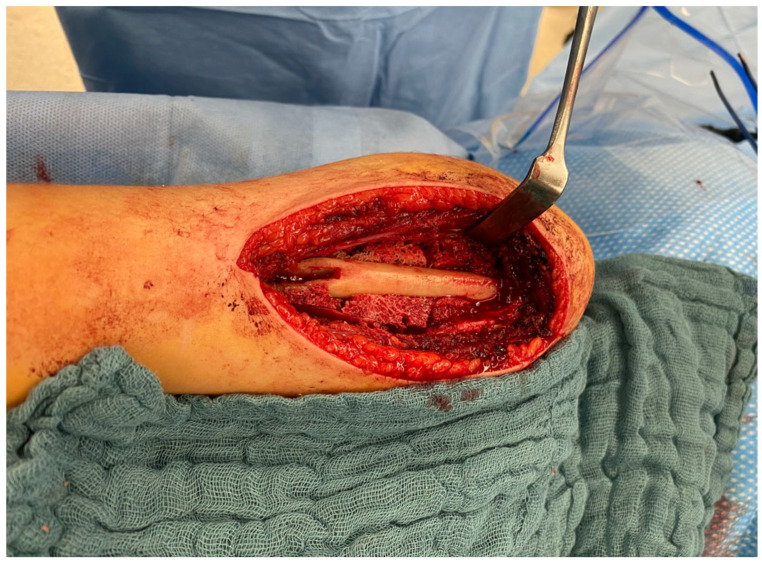
Post-reconstruction view of the lesion. No additional implant was required for stabilization.

Defect reconstruction is performed using an allogeneic fibula diaphysis graft (Deutsches Institut für Zell- und Gewebeersatz, DIZG, Berlin, Germany). The allogeneic fibula is cut to the required length, and a wedge-shaped distal end is prepared to allow intramedullary placement within the humeral diaphysis (Figure 5 and Figure 6). To stabilize the graft, allogeneic femoral head wedges (DIZG) are sculpted and tightly packed around the fibula within the humeral defect (Figure 5 and Figure 6).

Usually, no additional implants are required, as the graft is securely locked within the native bone. Finally, the periosteal sleeve is repositioned over the defect and sutured with PDS sutures, followed by standard wound closure.

#### 2.1.4. Postoperative Care

A Gilchrist bandage is recommended for four weeks and weight bearing is restricted for eight weeks. Radiological follow-ups are performed immediately postoperatively, at six and twelve weeks. Return to sports is allowed six months postoperatively, provided radiological consolidation is confirmed.

### 2.2. Methods

This retrospective study was approved by the institutional ethics committee (Ethik-Kommission des Fachbereichs Medizin der Goethe-Universität Frankfurt am Main 2021-552).

All patients diagnosed with an aneurysmal bone cyst of the proximal humerus and treated following the described protocol at our level I trauma center between January 2020 and December 2024 were identified and retrospectively reviewed.

Diagnosis was confirmed by clinical examination, conventional X-rays, and MR angiography. Furthermore, a histopathological analysis was performed for all cases.

Preoperative imaging (X-rays and MRI) was analyzed for the presence of pathological fracture, lesion size and extent, physeal involvement, and postoperative radiological consolidation. Cyst size was expressed both as absolute length (mm) and as a percentage of total humeral length to account for differences in patient age and bone size. Preoperative radiographs were classified according to the Campanacci classification [14]. For subgroup analysis, patients were stratified according to the occurrence of recurrence. A post hoc power analysis was performed to better contextualize the statistical findings.

Postoperative radiological follow-up included X-rays immediately after surgery, at six and twelve weeks.

Functional outcomes and range of motion were assessed for at least three months postoperatively. Patient data were anonymized before analysis.

Statistical analysis was performed to evaluate potential associations between prior surgery, cyst size, and recurrence. To compare recurrence rates between previously operated and non-operated patients, Fisher’s exact test was used. The strength of this association was further quantified using an Odds Ratio (OR) with a 95% confidence interval (CI).

To assess whether the percentage of humeral length affected by the cyst influenced the recurrence rate, an independent samples *t*-test was conducted to compare cyst expansion between patients with and without recurrence. Additionally, a Spearman correlation analysis was performed to examine the relationship between cyst size and recurrence. Statistical significance was set at *p* < 0.05 for all tests.

## 3. Results

### 3.1. Patient Characteristics

Between March 2020 and December 2024, a total of twelve patients were treated using the described technique. Two of them presented to us with a recurrent cyst and underwent other surgical treatment before (Patient 1 and 2, Table 1). The median age at the time of treatment was 9 years (range: 3–16 years). Eight patients were male, and four were female. The right humerus was affected in seven cases, while the left humerus was involved in five cases.

All patients presented with pathological fractures of the proximal humerus. The craniocaudal extent of the lesion ranged from 51 mm to 110 mm (median 76.5 mm), while the mediolateral extent varied between 17 mm and 58 mm (median 24 mm).

According to the Campanacci classification, four lesions were classified as Type 2 and seven as Type 4, one as Type 5 (no Type 1 or 3 lesions were observed).

In all cases, the lesion involved the proximal third of the humeral meta- and diaphysis with extension into the second third in eight patients. Growth plate involvement was observed in only one patient. Patient characteristics are displayed in Table 1.

MR angiography identified branches of the circumflex humeral artery as feeding vessel in eight patients. In one case, the lesion was supplied by the profunda brachii artery, and in three patients, no arterial feeders could be identified.

### 3.2. Histopathology

Histopathological analysis revealed fragmented bone tissue, cystic wall remnants, osteoclastic giant cells, and hemosiderophages, confirming the diagnosis of aneurysmal bone cyst (ABC).

### 3.3. Outcome

#### 3.3.1. Recurrence and Healing

The median follow-up period was 8.5 months (range, 3–48 months). Two of twelve patients (16.7%) experienced recurrence after treatment according to our protocol (Patients 2 and 5; Table 2). One of them had previously been treated with cyst filling using ChronOs without preoperative embolization.

**Figure 7:**
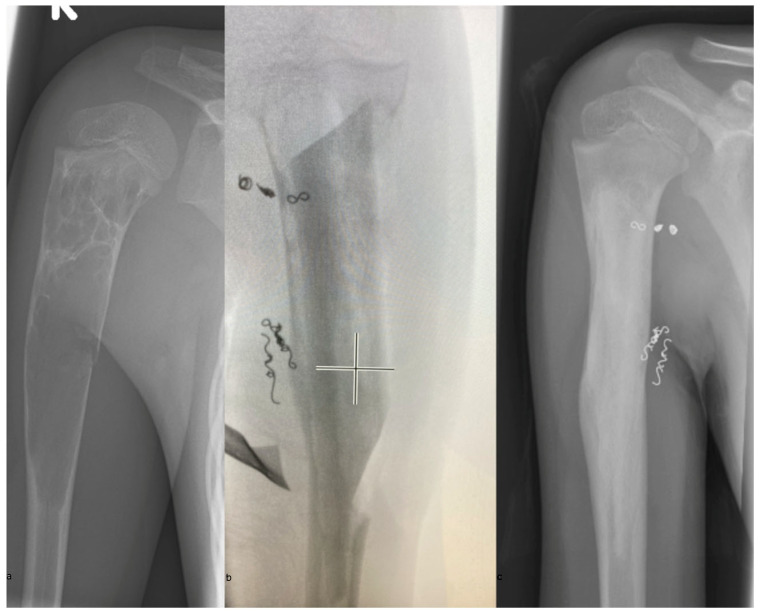
Preoperative X-ray, showing the expansion of the cyst and pathological fracture (**a**), intraoperative fluoroscopy after reconstruction (**b**) and complete consolidation of the humerus one year postoperatively (**c**).

Three patients (Patients 1, 2, and 5) were treated for recurrent cysts, two of whom had previously undergone different surgical treatment (Patients 1 and 2; without embolization, defect filling with ChronOs). Primary consolidation was achieved in 9 out of 12 patients (75%) after 5 months (median, 3–12 months) (Figure 7). Patients 1 and 5 required revision surgery but achieved final consolidation, resulting in an overall healing rate of 91.7% after secondary interventions (Table 2).

Analysis of the association between prior surgical intervention (other than SAE) and recurrence was performed using Fisher’s exact test. The test yielded a *p*-value of 0.045, which is just below the significance threshold of 0.05. This suggests a statistically significant difference in recurrence rates between previously operated and non-operated patients.

At final follow-up, healing was assessed according to the modified Neer classification. Eleven patients were classified as Grade I. One patient (Patient 2) showed persistent disease (Grade IV), and none as Grade II or III. Minor residual irregularities on radiographs were interpreted as remodeling changes without clinical relevance.

Patients with recurrence showed a larger mean cyst size compared to those without recurrence, both in absolute terms (90.3 ± 17.1 mm vs. 72.2 ± 17.8 mm) and when normalized to humeral length (40.3% ± 8.1% vs. 30.3% ± 8.7%). Although this difference did not reach statistical significance, a clear trend was observed. Median values showed a similar distribution between groups.

**Figure 8:**
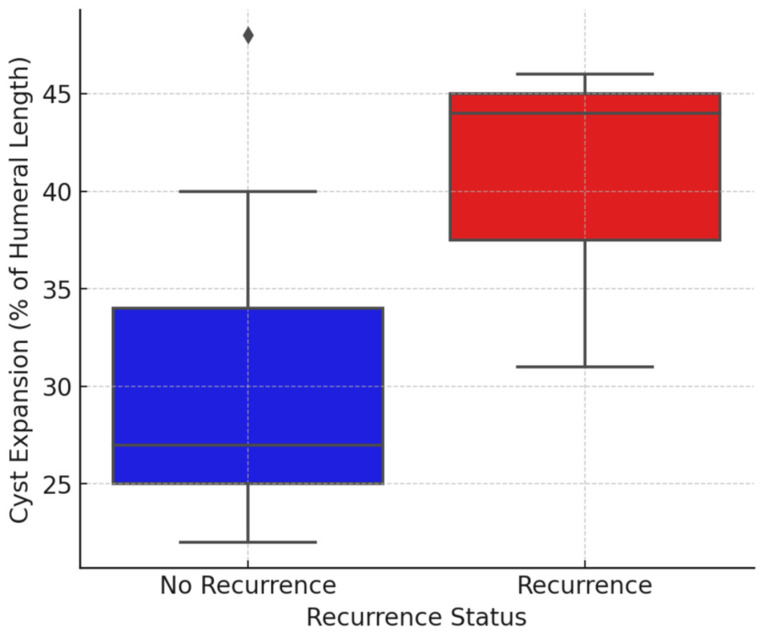
Comparison of cyst expansion between recurrence and non-recurrence group.

To assess whether the percentage of humeral length affected by the cyst influences the recurrence rate, a *t*-test for independent samples was performed. The test resulted in a *p*-value of 0.151, indicating no statistically significant difference in cyst expansion between patients with and without recurrence (Figure 8).

Additionally, a Spearman correlation analysis was conducted to examine the relationship between cyst size and recurrence rate. The correlation coefficient was r = 0.475, suggesting a moderate positive association, but the result was not statistically significant (*p* = 0.119). A post hoc power analysis revealed a statistical power of approximately 55% for detecting differences in cyst size between groups.

#### 3.3.2. Functional Outcomes

Excellent functional outcomes were observed in all but one patient. Patient 2 showed a slight limitation in range of motion (Anteflexion 160°, Abduction 130°).

No neurovascular deficits were noted in any patient, no shortening of the humerus was observed. Table 2 summarizes therapy and outcome parameters.

## 4. Discussion

This study evaluates a single-stage approach combining embolization, intralesional resection, and structural reconstruction for proximal humerus aneurysmal bone cysts in children. The main findings include a high rate of primary healing, reliable structural restoration, and a low recurrence rate.

Surgical curettage with or without bone grafting remains the gold standard for ABC treatment [3,7,15]. Various local adjuvants, including cryotherapy, phenol application, polymethylmethacrylate (PMMA), and doxycylin foam, have been introduced to reduce local recurrence rate [4,5,16]. However, recurrence rate for curettage and defect filling remains high, with rates up to 59% reported in the literature [12].

More aggressive approaches, such as intralesional high-speed burr debridement, have improved recurrence rates, with cure rates reaching 82% [17,18]. The use of argon beam coagulation has also demonstrated promising results [19], though potential risks include, e.g., osteonecrosis [20].

Radical en-bloc resection offers the lowest recurrence rates [15], but this comes at the cost of functional impairment, including pain, restricted range of motion, and muscle weakness [6,18].

Elastic intramedullary nailing (ESIN) has been described as a minimally invasive treatment option providing immediate mechanical stability and continuous decompression of cystic lesions, particularly in the presence of a pathological fracture. While this technique is well established in the treatment of unicameral bone cysts, its role in aneurysmal bone cysts remains less clearly defined and is not emphasized in current treatment concepts, which primarily focus on intralesional resection, sclerotherapy, or embolization [3].

In addition, combined approaches using ESIN in conjunction with curettage and defect filling have been reported [21], highlighting the need for supplementary structural support in selected cases.

In contrast, the present technique aims to achieve immediate biological and structural reconstruction of the defect without the need for permanent implant material. This may be particularly advantageous in larger lesions with substantial bone loss.

Selective arterial embolization (SAE) has been explored as standalone or adjunctive treatment, particularly for anatomically challenging ABCs (e.g., spine or pelvis), where complete surgical resection is difficult [3,5,18]. SAE alone has shown local control rate of 94%, but primary bone healing was observed in only 58.7% of cases, often requiring repeated embolization [5]. In isolated SAE treatment, up to 39% of cases required follow-up embolization [18].

Mostafa et al. reported good outcomes using a similar reconstruction technique involving a fitted fibula graft after resection, debridement with a high-speed burr, and argon beam coagulation. However, their approach involved autologous fibula grafting, which carries the risk of donor site morbidity [22]. By using allogeneic fibula grafts, we avoided this complication while achieving comparable healing outcomes without adverse effects.

In terms of functional outcome, our results were highly favorable; a total of 91.7% of patients regained full range of motion, with only one patient exhibiting minor motion restriction (Anteversion 160°, Abduction 130°). No cases of neurovascular deficits or humeral shortening were observed.

Primary consolidation was achieved after a median of 5 months (range, 3–12 months). Two patients were only available for follow-up for 3 months; however, both showed radiological signs of healing within that period. However, recurrence in our cohort occurred after a longer interval (13–36 months), highlighting the importance of extended follow-up to detect late-onset recurrence.

Recurrence remains a major concern in the treatment of aneurysmal bone cysts. In our cohort, two of twelve patients (16.7%) experienced recurrence after treatment according to our protocol (Patients 2 and 5; Table 2). Three patients in total were treated for recurrent cysts, two of whom had undergone previous surgical treatment using different techniques. Of these, one patient developed a further recurrence despite treatment according to our protocol. In a study of 150 ABC cases, Mankin et al. found no significant correlation between recurrence rate and patient age, sex, or lesion size, but reported a strong association with surgical technique [11].

In our analysis, a significant association was observed between prior surgery (using a different technique) and recurrence (*p* = 0.045, Fisher’s exact test). Odds Ratio (OR) was infinite, indicating a potentially higher risk in previously operated patients, though statistical certainty is limited due to the small sample size.

This association may reflect differences in the underlying characteristics of the lesions rather than the effect of the treatment itself. Patients with a history of previous surgery likely represent a selected subgroup with more complex or advanced lesions, which may be associated with a higher intrinsic risk of recurrence. In addition, prior procedures may alter the local bone environment, potentially making complete removal of cystic tissue more challenging. Further molecular studies, including the analysis of USP6 fusion partners, may help to better understand potential factors associated with recurrence.

Interestingly, we found no significant correlation between the cyst size (percentage of humeral length affected) and recurrence (*p* = 0.151, *t*-test). However, the observed trend towards larger cyst size in the recurrence group may indicate increased biological activity of the lesion. The lack of statistical significance despite a noticeable difference may be explained by limited statistical power due to the small sample size, as supported by the post hoc analysis.

Previous studies identified younger age (<10 years), juxta-epiphyseal location and female sex as potential risk factors for recurrence [5,23]. All recurrent cases in our cohort involved patients aged ≤ 10 years, but all were male.

The findings of this study provide a practical guideline for the surgical management of aneurysmal bone cysts (ABCs) of the proximal humerus, particularly in pediatric patients. The combination of preoperative SAE, intralesional resection, and allogeneic fibula grafting offers a minimally invasive yet effective approach, reducing intraoperative bleeding, minimizing recurrence rates, and preserving shoulder function. This protocol represents a valuable alternative to traditional curettage or radical en-bloc resection, especially in cases where preserving the growth plate is essential.

Given the favorable functional outcomes and acceptable recurrence profile observed in this cohort, this approach may be considered a valuable treatment option for selected pediatric patients with proximal humerus ABCs. Despite the limited sample size, the present cohort is clinically relevant because it represents a rare lesion in a highly specific anatomical location treated with a uniform single-stage protocol.

Additionally, its applicability in other anatomical regions warrants further investigations in future studies.

Emerging new treatment strategies including denosumab therapy—an anti-RANKL antibody used in aggressive bone lesions—offer intriguing prospects. However, severe hypercalcemia has been reported in up to 28% of cases, limiting its widespread use, and currently restricting it to a rescue therapy [4,16,24].

## 5. Conclusions

The combined approach of preoperative embolization, high-speed burr debridement, and structural allograft reconstruction addresses key limitations of conventional treatment by combining vascular control and mechanical stabilization in a single-stage procedure. In this cohort, the technique was associated with high healing rates, favorable functional outcomes, and an acceptable recurrence profile while avoiding donor site morbidity. This approach may represent a practical and reproducible treatment option for pediatric proximal humerus ABCs.

## 6. Limitations

This study has several limitations, including its retrospective design and small sample size. Despite the limited sample size, this study represents a homogeneous cohort of a rare pediatric condition treated with a standardized protocol at a single center. A major limitation is the relatively short follow-up period, with more than half of the patients having less than one year of follow-up. This may be insufficient to capture late recurrences and should be considered when interpreting the reported recurrence rates. Notably, recurrence occurred at 13 and 36 months in two cases, underscoring the importance of long-term follow-up to identify late recurrences.

Future prospective studies with larger cohorts and extended follow-up are required to confirm these findings and refine the treatment protocol.

## Figures and Tables

**Table 1:** Patient characteristics.

Pat. Nr	Sex	Age (Years)	Side	Localization (Thirds of Humerus)	% of Humeral Length	Length (mm)	Width (mm)	Growth Plate Involved	Campanacci Type	Previous Treatment
1	M	3	R	1–2	46	82	22	No	2	Curretage/ChronOs (10 months prior)
2	M	9	R	1–2	44	110	58	Yes	4	Curretage/ChronOs (39 months prior)
3	M	9	L	1	25	62	25	No	4	None
4	M	6	L	1	25	61	28	No	4	None
5	M	10	R	1	31	79	28	No	2	None
6	M	9	R	1	22	53	24	No	4	None
7	M	7	R	1–2	48	98	17	No	4	None
8	F	11	L	1–2	40	100	27	No	5	None
9	F	12	L	1–2	27	72	24	No	4	Cons. treatment of path. fracture, treated for juvenile bone cyst
10	M	4	R	1–2	29	51	21	No	4	Failed conservative treatment of path. fracture
11	F	16	R	1–2	23	78	23	No	2	None
12	F	7	L	1–2	34	75	18	No	2	None

**Table 2:** Details of treatment and outcome.

Pat. Nr	Arterial Feeder	Embol.	Transplant	Recurrence	Shortening/Angulation	Revision/Treatment	ROM	Consol. (Time to Consol. in Months)	Neer Class.	Follow Up (Months)
1	Ant. hum. circumflex	Coiling	1st surgery: ChronOs 2nd surgery: Fibula	after ChronOs filling without embolization	No/No	Fibula (following protocol)	Free	Yes (3)	I	7
2	Ant. hum. circumflex	Coiling	Fibula fem. head	1st after ChronOs filling without embolization 2nd recurrence after 36 months after protocol treatment	No/No	1st revision: Fibula (following protocol) No further revision after 2nd recurrence	Ante 160° Abd 130°	No (-)	IV	38
3	Ant./post. hum. circumflex	Coiling	Fibula	no	No/No	-	Free	Yes (4)	I	48
4	Ant. hum. circumflex	Coiling	Fibula fem. head	no	No/No	-	Free	Yes (4)	I	10
5	Ant. hum. circumflex	Coiling	Fibula fem. head	1st 13 months after protocol treatment, progress distally 2nd Varus (74°) proximal path. fracture	No/No	1st revision: “warm” fibula, plating + screw fixation/epiphysiodeses for varus correction 2nd revision: plate removal after 10 months 3rd revision: fibula + plate	Free	Yes (2)	I	3
6	Ant. hum. circumflex	Coiling	Fibula fem. head	no	No/No	-	Free	Yes (6)	I	23
7	Ant. hum. circumflex	Coiling	Fibula fem. head	no	No/No	-	Free	Yes (3)	I	36
8	No feeder found	-	Fibula fem. head	no	No/No	-	Free	Yes (3)	I	3
9	No feeder found	-	Fibula fem. head	no	No/17°	Additional plate osteosynthesis Plate removal after 19 months	Free	Yes (12)	I	26
10	Art. profunda brachii	Intraop clipping	Fibula fem. head	no	No/No	-	Free	Yes (6)	I	6
11	Ant./post. hum. circumflex/Art. profunda brachii	Coiling	Fibula fem. head	no	No/No	-	Free	Yes (5)	I	6
12	No feeder found	-	Fibula fem. head	no	No/No	-	Free	Yes (6)	I	6

## Data Availability

The datasets generated and analyzed during the current study are not publicly available due to institutional restrictions and patient confidentiality, but are available from the corresponding author on reasonable request.
